# Epidemic Levels of Drug Resistant Tuberculosis (MDR and XDR-TB) in a High HIV Prevalence Setting in Khayelitsha, South Africa

**DOI:** 10.1371/journal.pone.0013901

**Published:** 2010-11-15

**Authors:** Helen S. Cox, Cheryl McDermid, Virginia Azevedo, Odelia Muller, David Coetzee, John Simpson, Marinus Barnard, Gerrit Coetzee, Gilles van Cutsem, Eric Goemaere

**Affiliations:** 1 Burnet Institute, Melbourne, Australia; 2 Médecins Sans Frontières, Cape Town, South Africa; 3 Monash University, Melbourne, Australia; 4 City of Cape Town Health, Cape Town, South Africa; 5 University of Cape Town, Cape Town, South Africa; 6 National Health Laboratory Service, Cape Town, South Africa; 7 National Health Laboratory Service, Johannesburg, South Africa; University of Stellenbosch, South Africa

## Abstract

**Background:**

Although multidrug-resistant tuberculosis (MDR-TB) is emerging as a significant threat to tuberculosis control in high HIV prevalence countries such as South Africa, limited data is available on the burden of drug resistant tuberculosis and any association with HIV in such settings. We conducted a community-based representative survey to assess the MDR-TB burden in Khayelitsha, an urban township in South Africa with high HIV and TB prevalence.

**Methodology/Principal Findings:**

A cross-sectional survey was conducted among adult clinic attendees suspected for pulmonary tuberculosis in two large primary care clinics, together constituting 50% of the tuberculosis burden in Khayelitsha. Drug susceptibility testing (DST) for isoniazid and rifampicin was conducted using a line probe assay on positive sputum cultures, and with culture-based DST for first and second-line drugs. Between May and November 2008, culture positive pulmonary tuberculosis was diagnosed in 271 new and 264 previously treated tuberculosis suspects (sample enriched with previously treated cases). Among those with known HIV status, 55% and 71% were HIV infected respectively. MDR-TB was diagnosed in 3.3% and 7.7% of new and previously treated cases. These figures equate to an estimated case notification rate for MDR-TB of 51/100,000/year, with new cases constituting 55% of the estimated MDR-TB burden. HIV infection was not significantly associated with rifampicin resistance in multivariate analyses.

**Conclusions/Significance:**

There is an extremely high burden of MDR-TB in this setting, most likely representing ongoing transmission. These data highlight the need to diagnose drug resistance among all TB cases, and for innovative models of case detection and treatment for MDR-TB, in order to interrupt transmission and control this emerging epidemic.

## Introduction

There are an estimated 13,000 cases of multidrug-resistant tuberculosis (MDR-TB) emerging in South Africa each year [1]. These estimates are primarily based on a national survey performed in 2001, combined with routinely reported case numbers, and thus many believe this to be an under-estimation of the current situation. In addition, South Africa has a growing epidemic of extensively drug-resistant tuberculosis (XDR-TB) associated with high mortality among HIV infected individuals [2]. The continued emergence of MDR- and XDR-TB poses a significant threat not only to tuberculosis control but also to progress made in the expanded provision of antiretroviral treatment (ART) for HIV.

Drug-resistant tuberculosis (DR-TB) requires much longer and more costly treatment regimens than drug-susceptible tuberculosis, and in most high HIV prevalence settings, there is limited capacity for diagnosis. Thus, in many settings, few patients are diagnosed with DR-TB and even fewer receive adequate treatment. The HIV epidemic has driven dramatic increases in tuberculosis case notifications in southern Africa [3]. While expanding access to ART is expected to ultimately reduce tuberculosis case notifications, it may also contribute to the large pool of individuals with increased vulnerability to TB created by the HIV epidemic [4]. The convergence of these conditions: the high rate of tuberculosis prevalence, a vulnerable population and the existence of undiagnosed and untreated drug resistant tuberculosis create the potential for dramatically increasing epidemics.

To date, there have been limited data available on the prevalence of DR-TB in high HIV prevalence settings. Only 12 countries in the African region have conducted nationwide surveys since 2000, with few disaggregating by HIV status [1]. While a number of countries are planning national representative surveys, such surveys aimed at deriving nationwide estimates of DR-TB burden may mask pockets of extremely high tuberculosis drug resistance, particularly in settings with existing high rates of both HIV and tuberculosis. There is an urgent need to quantify the extent of the drug resistant tuberculosis epidemic in these settings in order to advocate for and develop strategies for control. This study aimed to assess the burden of tuberculosis drug resistance in a peri-urban setting in Khayelitsha Township outside Cape Town, South Africa.

## Methods

### Study setting

Khayelitsha is a high population density township situated 30 km from Cape Town with a population estimated at more than 500,000. Poverty and unemployment are high and the majority live in informal housing. In 2006, the prevalence of HIV among antenatal clinic attendees was 33% and close to 6,000 tuberculosis cases were notified in 2008, giving an estimated case notification rate of 1158/100,000/year (based on an estimated population of 500,000) [5], [6]. In response to increasing numbers of DR-TB cases seen in Khayelitsha clinics and poor patient outcomes, a pilot project to provide community-based care and treatment for DR-TB was initiated in 2007 [7]. The pilot project is implemented by Médecins Sans Frontières (MSF) in collaboration with the City of Cape Town and the Provincial Government of the Western Cape.

### Survey design

A cross-sectional survey among clinic attendees suspected for pulmonary tuberculosis was conducted in two large primary care clinics in Khayelitsha between May and November 2008. These clinics combined account for 50% of the TB case burden in Khayelitsha. Clinic attendees, aged 18 years and over, not currently receiving TB treatment and in whom tuberculosis was suspected clinically, were eligible to participate. The study was explained by clinic staff and written informed consent was obtained from each participant. The study was approved by the University of Cape Town Ethical Review Committee and by both the City of Cape Town and the Western Cape Province Health Department.

The desired sample size was determined separately for new and previously treated culture positive TB cases. Previous tuberculosis treatment was defined as 1 month or more of anti-tuberculosis treatment. Based on estimated proportions of MDR-TB of 2% and 4% respectively in South Africa [8], minimum sample sizes were 121 and 236 respectively (precision 2.5% and 5% alpha level). For logistical reasons and allowing for missing data, a target of 250 in each category was sought.

### Drug susceptibility testing

Two sputum samples were collected one hour apart and were transported the same day to the National Health Laboratory Service (NHLS) TB laboratory in Cape Town as per routine practice. Fluorescence sputum smear microscopy was performed on both sputum specimens in accordance with guidelines from the International Union Against Tuberculosis and Lung Disease (IUATLD) [9]. One specimen was cultured using the BACTEC MGIT 960 system (BD Diagnostics Systems, Sparks, MD). Positive cultures were confirmed as *Mycobacterium tuberculosis* complex using Ziehl-Neelsen staining and *p*- nitrobenzoic acid testing [10].

Resistance to rifampicin and isoniazid was determined on positive cultures using a rapid line probe assay (LPA) (Hain GenoType MTBDRplus) as previously trialled in this laboratory [11]. All subcultures were later transported to the NHLS laboratory in Johannesburg for conventional culture-based drug susceptibility testing to rifampicin, isoniazid, ethambutol, pyrazinamide, streptomycin, ofloxacin, ethionamide and the second-line injectable agents (amikacin, kanamycin and capreomycin) also using the BACTEC MGIT 960 system. Two concentrations of isoniazid were tested, 0.1 and 0.4 µg/ml, to assess high and low level isoniazid resistance [12]. For rifampicin and isoniazid, drug resistance was defined as resistance shown on either the rapid LPA or the conventional culture based susceptibility test. For isoniazid, low level resistance was defined as resistant. Multidrug resistant tuberculosis (MDR-TB) is defined as resistance to both isoniazid and rifampicin and extensively drug resistant tuberculosis (XDR-TB) is defined as MDR-TB with additional resistance to a fluoroquinolone and a second-line injectable agent.

### Data collection and analysis

Data on previous TB treatment, demographics, HIV status and antiretroviral treatment at the time of TB diagnosis were recorded routinely during the clinical assessment by a primary care nurse. Data on previous TB treatment was additionally verified among patients starting treatment through a medical record review. All data were entered on a database using Excel (Microsoft Office 2003). Data analysis, including multivariate logistic regression models, was conducted with SPSS (Release 17.0.0, 2008).

To investigate factors potentially associated with rifampicin resistance, both univariate and multivariate logistic regression analyses were conducted. Previous TB treatment was classified as either: new (not previously treated), the most recent TB treatment episode in 2007/08 (the survey was conducted between May and November 2008) or the most recent treatment episode prior to 2007. Factors significant or approaching significance (p = 0.05) on univariate analysis were entered into the multivariate logistic regression models. Factors were coded as categorical variables with missing data included as a category. All factors were entered as a block into multivariate logistic regression models and goodness of fit was assessed with the Hosmer and Lemeshow statistic [13].

The MDR-TB burden in Khayelitsha was estimated by applying the proportions of MDR-TB found through the survey to the case notification data for the whole of Khayelitsha in 2008 [6]. Estimated MDR-TB incidence was then calculated using an estimated population for Khayelitsha of 500,000. An approximation of MDR-TB transmission was made based on the assumptions that DR-TB among new cases represents transmission rather than acquired drug resistance and that the same level of primary transmission is likely to occur among previously treated TB cases, given extensive reinfection in endemic settings.

## Results

### Culture positive tuberculosis

**Figure 1:**
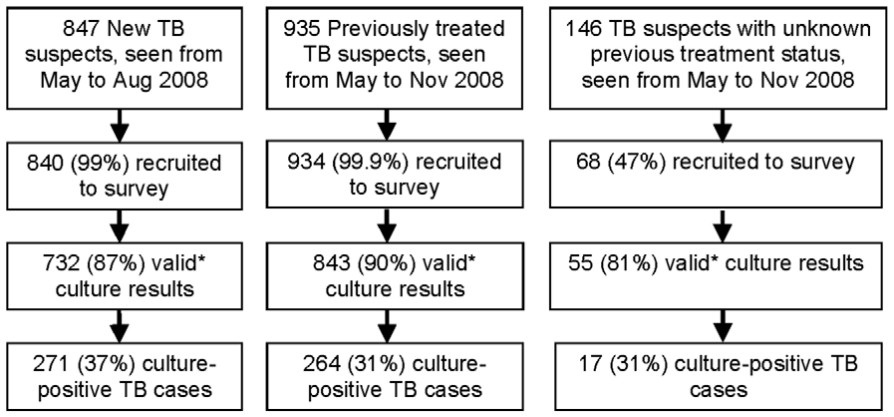
Participant recruitment and culture-positive tuberculosis diagnosed among TB suspects. New TB suspects were recruited from May through August, while previously treated suspects were recruited May through November, 2008. * Valid culture results include negative and positive cultures and exclude contaminated cultures, those found to be non-tuberculous mycobacteria or those with no growth.

During the study period, 1,842 (96%) of the 1,928 eligible clinic attendees suspected for pulmonary tuberculosis seen in the two clinics were recruited to the survey (Figure 1). Recruitment of participants not previously treated for tuberculosis ended in August 2008 as the desired sample size was estimated to have been reached. Recruitment of previously treated participants continued until November 2008, hence the overall combined sample does not reflect the relative proportions of new and previously treated TB suspects seen in the clinics.

In TB suspects for whom valid culture results were obtained (including valid positive and negative cultures), culture positive tuberculosis was diagnosed in 271/732 (37%) cases among those not previously treated and 264/843 (31%) cases among those with more than one month of previous tuberculosis treatment (Figure 1). The most common reasons for not obtaining a valid culture were contamination, non-tuberculosis mycobacteria and lost or leaked sputum samples. After investigation, 17 culture positive cases were excluded as the previous tuberculosis treatment status could not be determined (Figure 1).

HIV status was known for 88% of new and 90% of previously treated cases (Table 1). The most common reason for unknown HIV status was refusal to be tested. Among new TB cases with known HIV status, 55% were HIV infected, while 71% of previously treated cases were HIV positive (p = 0.001).

**Table 1:** HIV status, sex and age among new and previously treated culture positive TB cases (IQR =  interquartile range).

	New TB	Previously treated TB
Total	271	264
HIV negativeHIV positiveHIV status unknown	106132 (55% of known HIV status)33 (12%)	70168 (71% of known HIV status)26 (10%)
MaleFemale	155116 (43%)	16796 (36%)
Median age (IQR)Age 18–25Age 26–35Age 36+	32 (13)58 (21%)118 (44%)95 (35%)	36 (13)34 (13%)102 (39%)128 (48%)

### First-line anti-tuberculosis resistance

Valid LPA results were obtained for 267 (98.5%) of the 271 new TB cases and 259 (98.1%) of the 264 previously treated TB cases. Valid culture-based DST results were available for 237 (87.5%) and 221 (83.7%) of new and previously treated cases respectively (Table 2). Overall, resistance data (either LPA and/or culture DST) was available for 269 new and 261 previously treated TB cases (Table 2). Contamination of subsequent subcultures was the most common reason for missing culture DST results.

**Table 2:** Prevalence of drug resistance using line probe assay (LPA) results, conventional culture-based DST and combined among new and previously treated culture-positive TB cases.

	New TB cases	Previously treated TB cases
Total (positive culture)	271	264
LPA results available	267	259
Susceptible	244 (91%)	223 (86%)
H-mono	11 (4.1%)	7 (2.7%)
R-mono	4 (1.5%)	11 (4.2%)
MDR-TB	8 (3.0%)	18 (6.9%)
*Any Rifampicin resistance*	12 (4.5%)	29 (11.2%)
*Any Isoniazid resistance*	19 (7.1%)	25 (9.7%)
Culture-based DST available	237	221
Susceptible to first-line	186 (79%)	167 (76%)
First-line mono-resistance	35 (14.8%)	38 (17.2%)
H-mono	8 (3.4%)	15 (6.8%)
R-mono	2 (0.8%)	2 (0.9%)
First-line poly-resistance	9 (3.8%)	5 (2.3%)
MDR-TB Total	7 (3.0%)	11 (5.0%)
MDR-TB with second line resistance	3 (1.3%)	5 (2.3%)
XDR-TB	1 (0.4%)	1 (0.5%)
*Any Rifampicin resistance*	9 (3.8%)	13 (5.9%)
*Any Isoniazid resistance*	22 (9.3%)	31 (14.0%)
Either LPA and culture DST	269	261
MDR-TB	9 (3.3%)	20 (7.7%)
*Any Rifampicin resistance*	14 (5.2%)	29 (11.1%)
*Any Isoniazid resistance*	28 (10.4%)	41 (15.7%)

The line probe assay identified more rifampicin resistant cases than did culture-based DST, while more isoniazid resistance was identified through culture-based DST (Table 2). Overall, using both the LPA and culture-based DST results, 3.3% (9/269) and 7.7% (20/261) of new and previously treated cases were found to be infected with MDR-TB strains, with 5.2% (14/269) and 11.1% (29/261) infected with rifampicin resistant TB respectively (Table 2). Poly-resistance, most commonly isoniazid resistance combined with other first-line resistance apart from rifampicin was also frequently identified.

Table 3 compares the resistance profile from culture-based DST with that from the LPA for the 29 cases defined as MDR-TB. Of the 26 MDR-TB cases identified using the LPA, 7 were not able to be assessed with conventional culture-based DST, while 2 cases were defined as MDR-TB based on rifampicin resistance from the LPA and isoniazid resistance from conventional culture-based DST. A further MDR-TB case was susceptible using the LPA.

**Table 3:** Resistance profile (first and second-line) for all MDR-TB cases (abbreviations: H =  isoniazid, R =  rifampicin, E =  ethambutol, S =  streptomycin, Z =  pyrazinamide, Eto  =  ethionamide, Amk  =  amikacin, Km  =  kanamycin, Cm  =  capreomycin, Ofx  =  ofloxacin).

Resistance profile – culture-based DST	LPA MDR-TB	LPA Susc	LPA R-mono
no culture DST available	7		
Susceptible	1		
H	1		1
HR	2		
HRE	1	1	
HRS	1		
HREZ	3		
HRSZ	2		
HREZ Eto	1		
HRESZ Eto	1		
HZ Amk			1
HRS Km	1		
HRESZ Ofx	1		
HRESZ Eto Km Amk Cm	1		
HRESZ Eto Ofx	1		
HRESZ Eto Ofx Km Amk Cm	2		
Total	26	1	2

### Second-line anti-tuberculosis resistance

Among the 22 MDR-TB cases with culture-based DST available, 9 (41%) were found to have additional second-line resistance, including 7 (32%) with resistance to a fluoroquinolone or a second-line injectable agent or both (Table 3). Second-line resistance was also observed among strains with no first-line resistance and with mono- and poly-resistance to first-line drugs, most commonly resistance to ethionamide and capreomycin (Table 4).

**Table 4:** Second-line resistance among strains not defined as MDR-TB.

Resistance profile	Number
R Eto	1
H Eto	3
HS Eto	2
HZ Amk	1
Eto	7
Cm	6
Amk Cm Eto	1
Cm Ofx	1

### Estimating the burden of tuberculosis drug resistance in Khayelitsha

**Figure 2:**
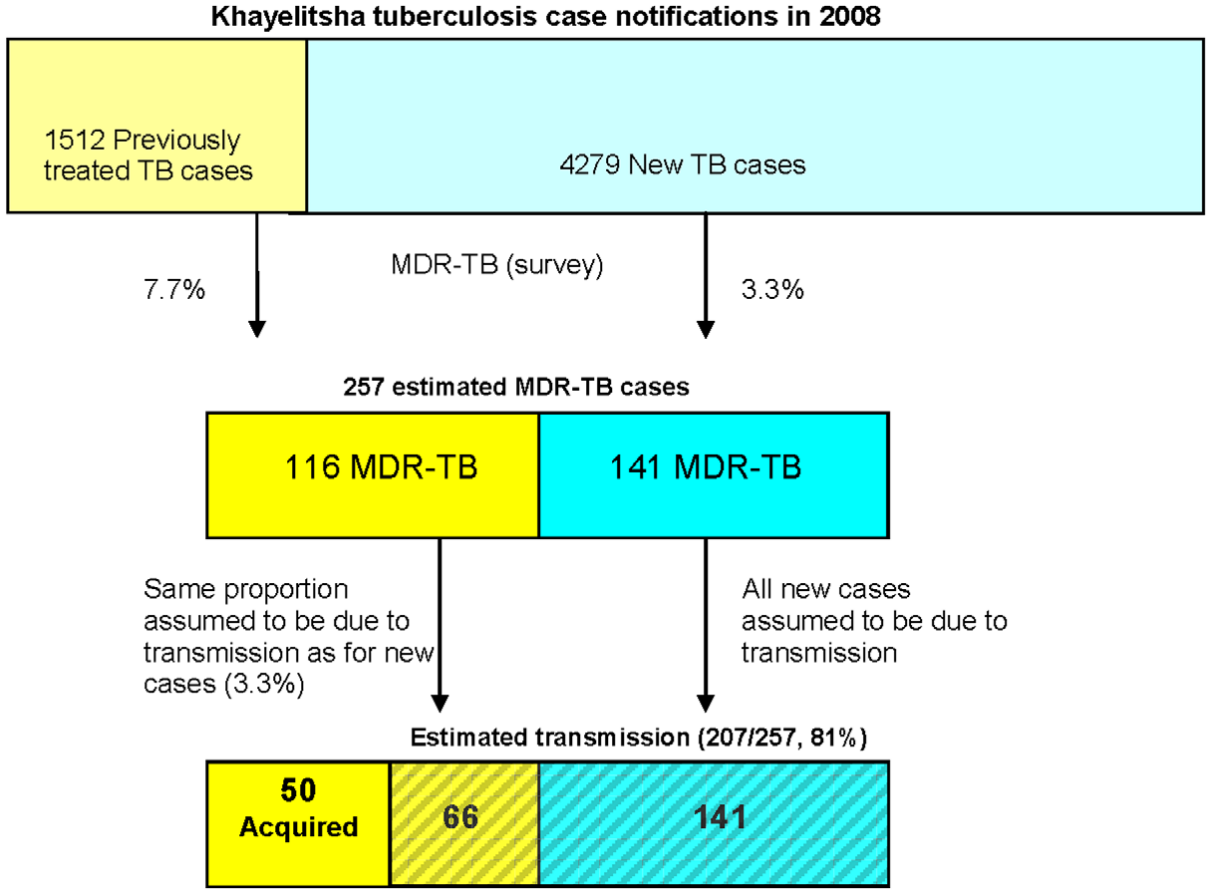
Estimating the burden of rifampicin resistant tuberculosis in Khayelitsha.

In 2008, 5,791 cases of pulmonary tuberculosis were reported from primary care clinics in Khayelitsha. When the percentages of MDR-TB are applied to these case notification figures, 257 MDR-TB cases would have been diagnosed if all TB cases were tested: 4.4% of the notified TB cases in that year (Figure 2). The majority (55%) would have been diagnosed among new, not previously treated TB suspects. These figures equate to an estimated notification rate of 51/100,000/year for MDR-TB, if all TB cases were to be tested. If MDR-TB among new cases represents direct transmission, then at least 141 (55%) of the estimated 257 MDR-TB cases are due to transmission. This proportion rises to 81% if we assume that a similar level of transmission occurs among the previously treated cases (Figure 2).

### Association between HIV infection, other factors and rifampicin resistant tuberculosis

Given the significant burden of rifampicin resistance not defined as MDR-TB, associations with HIV infection and other factors were assessed with rifampicin resistance. Among HIV infected new cases, 5.3% (7/131) were rifampicin resistant, compared to 3.8% (4/105) among HIV negative new cases. For HIV infected previously treated cases, 13.9% (23/165) were rifampicin resistant compared to 5.7% (4/70) among HIV negative cases. For the total combined sample, there was a significant association between HIV infection and rifampicin resistance on univariate analysis, but this did not reach significance in the multivariate analysis (OR = 2.26, 95% CI 0.91–5.61); a recent TB treatment episode and female sex were significant predictors (Table 5). As previous tuberculosis treatment is suggestive of drug resistance acquired under selective pressure of previous treatment, a separate multivariate model was constructed among previously treated TB cases only (Table 5). Similarly, the association with HIV infection did not reach significance (OR = 3.10, 95% CI 0.85–11.33) A recent TB treatment episode was the only significant factor associated with rifampicin resistance in this model, with female sex no longer significant. To further investigate associations between previous TB treatment, HIV infection and rifampicin resistance, a third multivariate model was constructed among HIV positive cases only (Table 5). Only a recent TB treatment episode was predictive of rifampicin resistance in this model.

**Table 5:** Univariate and multivariate analyses to assess associations with rifampicin resistance among all, previously treated, and HIV positive culture positive TB cases.

			Univariate			Multivariate		
Factor	Categories	Rifampicin resistant TB (%)	OR	95% CI	P value	OR	95% CI	P value
*Model includes all TB cases*								
Previous TB treatment	New (ref)Prev. TB 2006 or beforePrev. TB 2007/08Missing	14 (5.2%)10 (6.5%)15 (18.3%)4 (15.4%)	1.274.083.31	0.55–2.941.88–8.861.00–10.92	0.57<0.00010.049	1.404.424.23	0.59–3.321.97–9.931.22–14.59	0.45<0.00010.023
HIV status	Negative (ref)PositiveMissing	7 (4.0%)30 (10.1%)6 (10.2%)	2.712.71	1.16–6.300.88–8.44	0.0210.084	2.262.89	0.91–5.610.90–9.33	0.0790.075
Sex	Male (ref)Female	18 (5.6%)25 (12.0%)	2.30	1.22–4.33	0.010	2.03	1.01–4.06	0.047
Age	26+ (ref)18–25	31 (7.1%)12 (13.2%)	2.00	0.98–4.06	0.055	2.16	1.0–4.70	0.051
						*Hosmer and Lemeshow p = 0.166*		
*Model includes only previously treated TB cases*								
Most recent TB episode	2006 or bef (ref)2007/08Missing	10 (6.5%)15 (18.3%)4 (15.4%)	3.202.60	1.3–7.50.75–9.02	0.0070.13	3.423.02	1.43–8.150.85–10.80	0.0060.089
HIV status	Negative (ref)PositiveMissing	3 (4.3%)23 (13.9%)3 (11.1%)	3.622.91	1.1–12.50.55–15.46	0.040.21	3.103.17	0.85–11.330.57–17.5	0.0870.19
Sex	Male (ref)Female	13 (7.8%)16 (17.0%)	2.41	1.1–5.3	0.03	1.97	0.85–4.56	0.11
Age	26+ (ref)18–25	22 (8.7%)6 (18.2%)	1.98	0.74–5.30	0.17			
						*Hosmer and Lemeshow p = 0.273*		
*Model includes only HIV positive TB cases*								
Previous TB treatment	New (ref)Prev. TB 2006 or beforePrev. TB 2007/08Missing	7 (5.3%)7 (7.1%)14 (26.9%)2 (13.3%)	1.366.532.73	0.46–4.022.46–17.340.51–14.51	0.58<0.00010.24	1.677.274.26	0.53–5.232.61–20.250.74–24.37	0.38<0.00010.103
ART at TB diagnosis	No ART (ref)On ART at diagMissing	18 (8.7%)9 (17.3%)3 (7.9%)	2.190.89	0.92–5.200.25–3.20	0.0770.87	1.590.88	0.62–4.080.23–3.33	0.340.85
Sex	Male (ref)Female	10 (6.9%)20 (13.2%)	2.05	0.92–4.54	0.078	1.90	0.81–4.50	0.14
Age	26+ (ref)18–25	23 (8.9%)7 (18.4%)	2.31	0.92–5.82	0.077	2.18	0.75–6.33	0.15
CD4 at TB diagnosis	CD4 >100 (ref)CD4 <100CD4 missing	11 (8.5%)12 (15.8%)7 (7.8%)	2.030.91	0.84–4.860.34–2.45	0.110.86			
						*Hosmer and Lemeshow p = 0.991*		

## Discussion

Eastern Europe and the former Soviet Union have been described as global “hot spots” for tuberculosis drug resistance [14]. This has primarily been based on assessments of the *proportion* of all TB cases suffering from drug resistant disease. However a more accurate reflection of the burden imposed by MDR-TB and the threat of increasing spread can be given through assessing population based *incidence* rather than proportions [15]. Using this approach, South Africa has an estimated population incidence of MDR-TB similar to that in the Russian Federation; 26/100,000/year and 27/100,000/year respectively [1].

The data presented here show that in Khayelitsha, a densely populated urban township in South Africa, the estimated burden of MDR-TB is extremely high, at 51/100,000/year based on notified TB cases, with incidence likely to be considerably higher taking into account incomplete tuberculosis case detection in the community. A large proportion of MDR-TB cases have pre-existing second-line tuberculosis resistance and indeed XDR-TB in the absence of extensive previous second-line treatment (data not shown). These data suggest a burden of MDR-TB more than twice that previously estimated for South Africa. Given the high population density, poverty, high HIV and TB prevalence in Khayelitsha, these data are unlikely to be representative nationally. However the living conditions in Khayelitsha are reflective of the large proportion of South Africans who are most at risk for tuberculosis. Urban and peri-urban townships like Khayelitsha therefore should be the focus of particular attention for both surveillance and epidemic control.

Currently available diagnostics for tuberculosis drug resistance rely on culture and are therefore slow, cumbersome and commonly only available through centralised laboratories. As a result, DST is only selectively available if at all and even in South Africa is only available for previously treated TB cases. The data from Khayelitsha suggest that a large proportion of MDR-TB cases occur in new TB suspects, who are not routinely tested for drug resistance. These patients are therefore likely to receive ineffective first-line anti-tuberculosis treatment resulting in likely amplification of resistance, poor treatment outcomes including increased mortality, and not least fuelling increasing transmission through remaining infectious and inadequately treated in the community. If DST is only accessible for those failing treatment, a large proportion of cases will be missed due to the high risk of death among HIV positive patients with untreated DR-TB [2]. Early diagnosis and improved case detection, through increasing access to DST, either with culture or newer diagnostics such as the line probe assays, is therefore fundamental to both identifying the scale of this epidemic and to developing strategies for control, in addition to reducing mortality.

The extent of MDR-TB among new, previously untreated TB cases in this survey suggests that at least half of all MDR-TB is due to ongoing primary transmission in Khayelitsha. Transmission may be even higher; up to 80% of cases may be transmitted, as it cannot be assumed that MDR-TB among previously treated cases is always due to resistance amplification. While this is a gross approximation of transmission, clearly transmission remains a substantial cause of incident MDR-TB cases in this setting.

Tuberculosis drug resistance has traditionally been blamed on poor TB control programmes and poor patient compliance with treatment. However, HIV infection has also been suggested to contribute to both an increased risk of acquiring resistance and to the risk of direct infection with DR-TB. Acquired rifamycin resistance has been demonstrated among HIV positive TB patients in well controlled clinical trials and other studies [16]–[18]. Contributing factors in these studies include low CD4 levels, co-administration of ART, extra-pulmonary TB and treatment for co-morbidities. Drug malabsorption has been demonstrated among HIV positive patients receiving tuberculosis drugs [19], [20], but there is limited data among those receiving concomitant ART. Given that in high TB prevalence areas, HIV positive individuals, even those receiving ART, are likely to repeatedly develop active tuberculosis and receive treatment [21], the development of DR-TB, independent of poor treatment adherence and other programme factors can be hypothesised.

HIV infection was not significantly associated with rifampicin resistant TB in multivariate analyses, either overall or among previously treated TB cases in this large community based representative survey. Rather, consistent significant associations were found between recent TB treatment and rifampicin resistance. These data suggest that any observed associations between HIV infection and DR-TB may be mediated through the propensity for HIV infected individuals to be repeatedly treated for TB and that HIV is not an independent risk factor for infection and development of active DR-TB disease. However, without prospective data and molecular genotyping of strains from subsequent TB episodes, it is not possible to differentiate the extent of acquisition of resistance during TB treatment, and the impact of HIV infection and concomitant ART on resistance acquisition, from reinfection with a DR-TB strain. It may be that HIV infected individuals who have been treated for TB previously are more exposed to DR-TB in nosocomial settings. Indeed, nosocomial transmission is suggested to be a significant cause of MDR- and XDR-TB transmission in Msinga district, KwaZulu Natal, South Africa [22], while previous hospitalisation was also an independent predictor for MDR-TB among previously treated TB cases in the South African national prevalence survey [8].

This survey is subject to the usual limitations in survey design and data collection. While restricted to two primary care clinics in Khayelitsha, these two clinics were responsible for diagnosing and treating approximately 50% of TB cases in Khayelitsha in 2008. Nonetheless, if nosocomial transmission is a significant factor driving DR-TB transmission, the risk of DR-TB infection through a larger clinic may be greater than that in the smaller primary care clinics in Khayelitsha. While previous tuberculosis treatment was verified through medical record review when possible, there is likely to be a tendency for patients to not report previous treatment in order to avoid receiving daily streptomycin injections and the longer treatment course. While such a bias could lower our estimate of overall MDR-TB prevalence, the data still suggest a substantial number of MDR-TB cases would be diagnosed if all TB cases were to be tested.

The Khayelitsha tuberculosis treatment programme reports reasonable treatment outcomes for 2008; currently treatment success stands at 82% for new TB cases and 50% for previously treated cases [6]. However this is a relatively recent improvement, with outcomes prior to 2003 significantly lower than this, including substantial rates of treatment interruption and default. Such conditions are likely to have contributed to the initial development of acquired resistance among patients receiving poor tuberculosis treatment [23]. However, the data presented here suggest that while acquired resistance may remain important, the great majority of MDR-TB is now due to direct transmission of already resistant tuberculosis strains. The overall lack of association with HIV infection is therefore unsurprising.

Although HIV infection, along with previous sub-optimal tuberculosis treatment programmes, may have contributed to the initial emergence of tuberculosis drug resistance, the high community HIV prevalence in Khayelitsha does not appear to be a significant factor *selectively* driving DR-TB transmission in this setting. Rather high HIV prevalence is driving transmission of both drug susceptible and drug resistant tuberculosis. High rates of transmission of drug resistant tuberculosis highlight the need for improved infection control measures, both in health care facilities and at community level. Based on the large burden of DR-TB in this setting and given that it is likely that the epidemic is being driven by transmission, innovative models of care aiming to diagnose and treat as many DR-TB cases as possible and as early as possible will be required to interrupt transmission. Such models will by necessity require rapid DR-TB diagnostics as close to point of care as possible, and for all TB suspects, along with decentralised treatment of patients. It is unlikely that any centralised system will be able to cope with the large numbers of patients requiring treatment or to hold them on treatment for the full time required. Existing tuberculosis treatment services will need to be utilised and strengthened in order to expand access to diagnosis and treatment for drug resistant TB.
